# Effects of URB937 on an animal model of migraine pain

**DOI:** 10.1186/1129-2377-14-S1-P68

**Published:** 2013-02-21

**Authors:** R Greco, AS Mangione, R De Icco, T Bandiera, G Sandrini, G Nappi, D Piomelli, C Tassorelli

**Affiliations:** 1 Headache Science Centre, IRCCS National Neurological Institute “C. Mondino” Foundation , Pavia, Italy; 2 Medicinal Chemistry Drug Discovery and Development, Italian Institute of Technology, Genova,Italy; 3 Headache Science Centre, IRCCS National Neurological Institute “C. Mondino” Foundation , UCADH Pavia, Italy; 4 Departments of Pharmacology and Biological Chemistry, University of California, Irvine, CA, USA

## 

Several studies have suggested the existence of interactions between the endocannabinoids and migraine. URB937, a FAAH inhibitor specific to peripheral tissues, causes analgesia in animal models of pain [1]. In this study, we evaluated whether the URB937 administration may alter nociceptive responses in an animal model of migraine based on nitroglycerin (NTG)-induced hyperalgesia [2]. Rats received systemic NTG and URB937 before being evaluated at the Tail flick test or at the Formalin test. The findings show that URB937 did inhibit NTG-induced hyperalgesia at the Formalin test with only a minimal influence on the hyperalgesia at the Tail flick. The data suggest that availability of anandamide probably at the meningeal level is effective in the migraine pain.
